# Bilateral Hypertrophic Olivary Degeneration Following Unilateral Mesencephalic Hemorrhage

**DOI:** 10.1002/ccr3.72005

**Published:** 2026-02-03

**Authors:** Saeed Razmeh, Amir Mohammad Dashti, Amir Hassan Habibi

**Affiliations:** ^1^ Neurology Department Yasuj University of Medical Science Yasuj Iran; ^2^ Student Research Committee Yasuj University of Medical Sciences Yasuj Iran; ^3^ Department of Neurology, Rasoul Akram Hospital Iran University of Medical Sciences Tehran Iran

**Keywords:** Claude's syndrome, dentato‐rubro‐olivary pathway, hypertrophic olivary degeneration, mesencephalic hemorrhage, palatal myoclonus

## Abstract

After a unilateral midbrain hemorrhage, clinicians should recognize that bilateral hypertrophic olivary degeneration may occur, presenting as palatal myoclonus, dysarthria, or tinnitus. This highlights the necessity for timely diagnosis and effective symptom management.

A 65‐year‐old man with a history of hypertension and smoking presented with bilateral auditory clicking. Two months earlier, he had presented with Claude's syndrome, including left oculomotor nerve palsy, right hemiataxia, and mild right hemiparesis. Computed tomography scan of the brain revealed a hyperdense lesion in the midbrain (Figure 1, arrow), suggestive of hemorrhagic stroke, and was treated conservatively. The current neurologic examination revealed right hemiataxia, accompanied by slurred speech and a consistent, bilateral, rhythmic, jerky movement of the soft palate, which indicated bilateral palatal myoclonus (Video 1). Magnetic resonance imaging (MRI) of the brain revealed hypertrophic degeneration of both inferior olivary nuclei on transverse T2‐weighted sequence and fluid‐attenuated inversion recovery (FLAIR) sequence (Figure 2, arrows). Palatal myoclonus (also known as palatal tremor) is characterized by rhythmic, involuntary contractions of the muscles of the soft palate. Bilateral palatal myoclonus involves synchronous rhythmic contractions of both sides of the soft palate. The disorder can present with symptoms such as objective tinnitus (clicking sounds audible to both patient and examiner), dysarthria, and, in rare cases, dysphagia, depending on the extent of muscle involvement [1]. Palatal myoclonus caused by degeneration of the inferior olivary nucleus refers to a movement disorder characterized by rhythmic, involuntary contractions of the soft palate, resulting from a specific type of trans‐synaptic degeneration known as hypertrophic olivary degeneration (HOD). This condition arises after lesions that disrupt the dentato‐rubro‐olivary pathway (the Guillain‐Mollaret triangle), which connects the contralateral dentate nucleus of the cerebellum, the red nucleus, and the ipsilateral inferior olivary nucleus in the medulla [2]. Treatment is individualized and may include pharmacologic agents (e.g., benzodiazepines, anticonvulsants), botulinum toxin injections targeted to the involved muscles, or supportive measures for associated symptoms [3].

**FIGURE 1:**
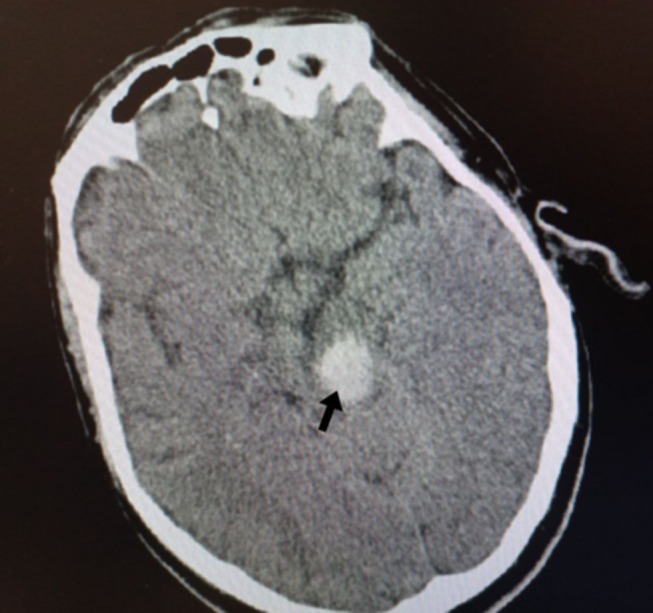
Axial computed tomography scan of the brain shows relatively hyperdensity in unilateral mesencephalic in favor of hemorrhage (arrow).

**VIDEO 1:** A 65‐year‐old man with a history of hypertension presented with right hemiataxia and speech impairment following a hemorrhagic stroke. Neurological examination revealed bilateral palatal myoclonus, characterized by constant, rhythmic jerky movements of the soft palate with an audible clicking. Video content can be viewed at https://onlinelibrary.wiley.com/doi/10.1002/ccr3.72005.

**FIGURE 2:**
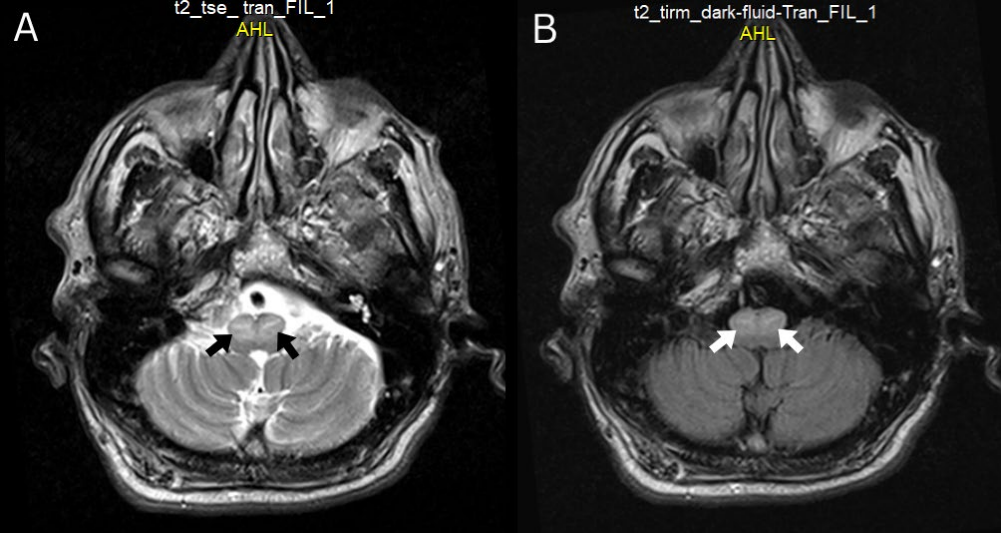
Axial T2‐weighted sequence (A) fluid‐attenuated inversion recovery (FLAIR) sequence (B) showing bilateral hypertrophic olivary degeneration (arrows).

## Author Contributions

**Saeed Razmeh:** conceptualization, data curation, supervision, writing – review and editing. **Amir Mohammad Dashti:** methodology, validation, writing – original draft, writing – review and editing. **Amir Hassan Habibi:** writing – review and editing.

## Funding

This work was supported by the Research Council of Yasuj University of Medical Sciences. The funding source had no role in the study design; in the collection, analysis, and interpretation of data; in the writing of the report; or in the decision to submit the article for publication.

## Ethics Statement

The protocol of this study was approved by the Institutional Ethics Committee of Yasuj University of Medical Sciences.

## Consent

Written informed consent was obtained from the patient for publication of the clinical details and accompanying images.

## Conflicts of Interest

The authors declare no conflicts of interest.

## Data Availability

The data that support the findings of this study are available from the corresponding author upon reasonable request.
